# Demography–environment relationships improve mechanistic understanding of range dynamics under climate change

**DOI:** 10.1098/rstb.2022.0194

**Published:** 2023-07-17

**Authors:** A.-K. Malchow, F. Hartig, J. Reeg, M. Kéry, D. Zurell

**Affiliations:** ^1^ Institute for Biochemistry and Biology, University of Potsdam, 14469 Potsdam, Germany; ^2^ Theoretical Ecology Lab, Faculty of Biology and Pre-Clinical Medicine, University of Regensburg, 93053 Regensburg, Germany; ^3^ Swiss Ornithological Institute, 6204 Sempach, Switzerland

**Keywords:** attribution science, individual-based model, Bayesian calibration, spatially explicit process-based model, range shifts

## Abstract

Species respond to climate change with range and abundance dynamics. To better explain and predict them, we need a mechanistic understanding of how the underlying demographic processes are shaped by climatic conditions. Here, we aim to infer demography–climate relationships from distribution and abundance data. For this, we developed spatially explicit, process-based models for eight Swiss breeding bird populations. These jointly consider dispersal, population dynamics and the climate-dependence of three demographic processes—juvenile survival, adult survival and fecundity. The models were calibrated to 267 nationwide abundance time series in a Bayesian framework. The fitted models showed moderate to excellent goodness-of-fit and discriminatory power. The most influential climatic predictors for population performance were the mean breeding-season temperature and the total winter precipitation. Contemporary climate change benefitted the population trends of typical mountain birds leading to lower population losses or even slight increases, whereas lowland birds were adversely affected. Our results emphasize that generic process-based models embedded in a robust statistical framework can improve our predictions of range dynamics and may allow disentangling of the underlying processes. For future research, we advocate a stronger integration of experimental and empirical studies in order to gain more precise insights into the mechanisms by which climate affects populations.

This article is part of the theme issue ‘Detecting and attributing the causes of biodiversity change: needs, gaps and solutions’.

## 1. Introduction

Changing climatic conditions are impacting natural systems around the globe, causing rapid biodiversity changes [1–3]. Two of the most striking and commonly discussed impacts are distribution shifts [4,5] and changes in population abundances [6,7]. These range dynamics result from an interplay of key ecological processes such as local population dynamics and dispersal, which are widely considered to be influenced by the environment [8,9]. An improved, model-based understanding of how climate affects range and population dynamics through these key processes may help to better explain and predict the observed responses [10,11]. Such insights are prerequisite for a quantitative, science-guided basis for deriving effective conservation measures to mitigate biodiversity loss [12].

A process-based approach to species distribution modelling has been suggested repeatedly, going beyond purely correlative models [13–16]. It is expected that process-based models can provide more reliable predictions under changing conditions by explicitly including causal eco-evolutionary mechanisms [8,17] and allowing for the representation of transient dynamics. Progress towards this goal was made with hybrid models that couple a phenomenological habitat model with processes like population dynamics and dispersal [18]. However, hybrid models do not allow an explicit link between demographic processes and the environment to be established. Instead, they assume that demography scales with a habitat suitability index, that is typically derived from a correlative model and may combine multiple land cover and climate variables [19]. Yet, the relation of such suitability measures to abundance or growth rate has been questioned [20,21]. Alternatively, a direct causal link can be established by considering explicit responses of processes, such as demography, to environmental predictors. For example, Schurr *et al*. [22] proposed a spatially explicit process-based model that considers parametric demography–environment relationships together with mechanistic dispersal effects.

Direct measurements of demography–environment relationships can be obtained by measuring demographic rates over an environmental gradient, which requires large-scale and well-designed monitoring schemes [23]. This has been done for a number of plants [24,25], but only rarely for animal species, for example fish (brown trout, [26]), or birds (North-American forest birds [27] and Arctic sea ducks [28]). As an alternative to direct measurements, Pagel & Schurr [29] suggested an inverse modelling approach that simultaneously estimates the demography–environment relationships and all other process parameters from empirical data. In the original formulation, they assumed a logistic growth (as the Ricker model) to describe local population dynamics [29]. Yet, a benchmarking study based on simulated data suggested that dynamic models with more complex life histories improved predictions of range dynamics [30]. Such an extension can be achieved with a refined population model that does not use a compound growth rate but considers explicit demographic sub-processes, such as survival and fecundity, together with their respective environmental responses.

Individual-based models (IBMs [31,32]) provide a flexible modelling framework that can accommodate such complex life histories by considering relevant demographic processes at the scale of individuals. We thus regard IBMs as ideal candidates for achieving the necessary flexibility [30]. Here, we extend the statistical framework introduced by Pagel & Schurr [29] to IBMs and jointly infer demography–environment relationships and dispersal for initially nine Swiss breeding bird populations from long-term abundance data. To this end, we explicitly modelled the demographic sub-processes of juvenile survival, adult survival and fecundity together with dispersal by single-species IBMs. All our models were built using RangeShifter [33,34], a modular IBM platform for simulating spatially explicit, eco-evolutionary dynamics that can be generically applied to different species. Within a Bayesian framework, we calibrated the IBMs to abundance time series from 267 sites across Switzerland that span the last two decades. In this period, complex range and population dynamics have been observed for Swiss birds [35,36]. Mountainous regions like the European Alps are particularly susceptible to current climate change, and altered temperature and precipitation patterns are already being observed [37]. In Switzerland, the detected trend in air temperature increase over five decades (1959–2008) reached 0.35 K/decade, which amounts to about 1.6 times the Northern Hemispheric warming rate [38]. We therefore expected that the observed range and population dynamics in Swiss birds are attributable to the climatic changes of the past decades.

Our goal was to assess if process-based models are able to provide useful predictions under changing climatic conditions and if they allow inference on the underlying mechanisms. For this, our models related different climate layers, which summarized key climatic variables during decisive periods of the year, directly to the spatio-temporal variation in demographic rates. We refer to these relationships as demography–climate relationships (DCRs), since we considered only climatic predictors. We examine the fitted DCRs for patterns across species and point out potential limitations in their causal interpretation, that originate from our data-driven calibration approach. To evaluate the DCRs, we map the demographic rates (juvenile survival, adult survival, fecundity) as well as the resulting local growth rate across Switzerland. Based on the calibrated model, we assess the impact of two decades of contemporary climate change on both the growth rate as well as on the abundance of each species. Such insights can facilitate the communication of severe consequences of climate change as well as the design of potential mitigation measures, targeting the specific demographic processes that are most impacted. Our approach is applicable to any population for which spatio-temporal abundance data are available. It can be flexibly extended to allow more detailed conclusions by incorporating more complex DCRs and using more fine-grained predictors.

## 2. Material and methods

### (a) Study area and data

Switzerland features strong elevational gradients, as large parts are located in the European Alps. The warming rates due to climate change show high spatial and seasonal variance, with their peak in summer at 0.46 K/decade and large values in the lowlands during autumn and in middle and high elevations in spring [38]. To describe the climatic variation over this landscape, we used bioclimatic data from CHELSA v. 2.1 [39,40]. It provides monthly means of daily minimum, mean and maximum temperatures, as well as total precipitation for the years 1901–2019 at a spatial resolution of 30 arcsec (≈1 km). These climate layers were averaged over several months for the breeding season (April–July), autumn (September–November) and winter (December–February), and standardized using the mean and standard deviation over the considered set of years (1997–2019). We further used land cover data from the CORINE project [41] for 2000, 2006, 2012 and 2018 at a resolution of 100 m to inform species-specific maps of suitable habitat (based on published information on habitat preferences, see below). Parameter inference was based on data from the standardized Swiss breeding bird survey (MHB) for the years 1999–2019. The MHB produces yearly time series at 267 sites selected in a systematic-random sampling design [42]. Each site comprises a 1 km^2^ square in which the number of breeding pairs was counted during two or three repeat surveys per year using the so-called simplified territory mapping method [42]. To develop models of initial abundance, we further used Atlas data of the period 1993–1996 [43] that provides a snapshot of counts at 2318 sites randomly distributed across Switzerland.

### (b) Model building and calibration

We selected a list of bird species according to a set of common characteristics, which allowed us to use the same model structure for all. We chose passerines that prefer forests, shrubs and mountainous regions as their main habitats, are sedentary or short-distance migrants, have a similar life history in which 1-year-olds can be considered mature and able to reproduce, and show changes in spatial abundance between the two Atlas periods of 1993–1996 and 2013–2016. Focusing on forest and upland species meant that effects of land use change such as intensification of agriculture are less likely to contribute substantially to population dynamics [44]. By excluding long-distance migrants, we could assume that local winter conditions are meaningful predictors of demographic rates. These criteria allowed us to isolate the effects of climate on the population dynamics as much as possible. Further constraints were imposed in order to keep the parameter calibration feasible: the relatively simple life history was chosen to limit the number of demographic model parameters and a minimum number of 170 non-zero counts in the MHB data was required. Overall, the criteria related to the species’ ecology and technical aspects of the parameterisation were fulfilled for nine species: Eurasian bullfinch, European crested tit, Eurasian treecreeper, Eurasian nuthatch, dunnock, goldcrest, common linnet, ring ouzel and alpine accentor.

**Figure 1.**
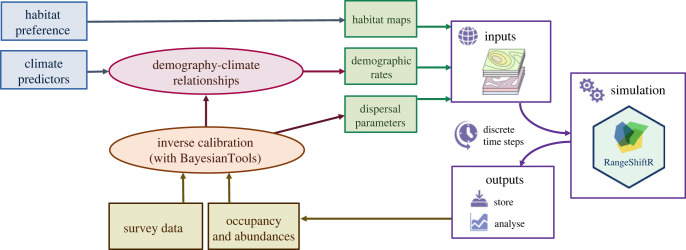
Model workflow: the RangeShifter model (right) has several inputs (green boxes): (1) habitat maps that are generated from habitat preferences (based on expert knowledge), (2) maps of demographic rates that are derived from demography–climate relationships (DCRs) and climate variables and (3) dispersal parameters. The DCR parameters and dispersal parameters are estimated inversely in a Bayesian calibration, comparing observed survey data and simulated abundances (yellow boxes).

Despite their common characteristics, we expected that the demography of the species will respond differently to climate variation [45,46]. To understand these responses, we built species-specific IBMs with the RangeShifter modelling platform [33], operated via the RangeShiftR R package [34]. The IBMs simulate the population and dispersal dynamics of each species on a regularly gridded landscape of Switzerland. We modelled population dynamics with a female-only, two-stage model comprising the stages ‘juvenile’ and ‘breeding adult’, with a transition time of 1 year between the two stages. The local population dynamics of this model are characterized by three demographic rates: juvenile and adult survival probability, *s*_*j*_ and *s*_*a*_, and fecundity *ρ*. These demographic rates are considered to be directly and independently influenced by the local climatic conditions and are thus allowed to vary spatio-temporally, i.e. among cells and years. This link between demography and climate is described by six DCRs (table 1). The coefficients of the DCRs as well as all other model parameters are inversely estimated for each species from MHB survey data within a Bayesian calibration (figure 1).

**Table 1.** Aggregated climatic predictors and their responses as modelled by the demography–climate relationships (DCRs). The climatic variables of mean temperature, minimum temperature and total precipitation were averaged over a relevant season and, up to the given order, related to a demographic rate as response variable. These responses include fecundity *ρ*, juvenile survival *s*_*j*_ and adult survival *s*_*a*_. The bracketted response was originally considered, but excluded due to high collinearity of *T_wn_* and *T_at_*.

climatic predictor	season	abbr.	order	response
mean temperature during breeding season	April–July	*T*_*br*_	2	*ρ*
total precipitation during breeding season	April–July	*P*_*br*_	2	*ρ*
mean temperature during autumn	Sep–Nov	*T*_*at*_	2	*s*_*j*_
minimum temperature during winter	Dec–Feb	*T*_*wn*_	1	(*s*_*j*_), *s*_*a*_
total precipitation during winter	Dec–Feb	*P*_*wn*_	2	*s*_*j*_, *s*_*a*_

To formulate the DCRs, each demographic rate was related to relevant climate predictors using the structure of a generalized linear model, with a logarithmic link function for fecundity *ρ* and a logistic link for survival, *s*_*j*_ and *s*_*a*_. As predictors, we considered mean or minimum temperature and total precipitation, averaged over the period of the year that was considered most relevant for the respective process (table 1): fecundity depended on the conditions during the breeding period (April–July); survival probabilities depended primarily on the winter conditions (December–February), with juvenile survival additionally affected by conditions in autumn (September–November) when juveniles are independent and are known to suffer from high mortality. All predictors were included with linear and quadratic polynomial effects, apart from minimum winter temperature, which was only included in linear form. Minimum winter temperature was highly correlated with autumn temperature (≈0.76), and was therefore excluded as predictor for *s*_*j*_ (but still included for *s*_*a*_). Therefore, the DCR of temperature on juvenile survival should be interpreted as the combined effects from autumn and winter temperature. Within the IBM simulation, the realized values of all demographic rates are then obtained from the calibrated, species-specific DCRs, using only the climate layers as input (figure 1).

In our model, survival probability describes the annual mortality that primarily occurs during winter and additionally during autumn for juveniles. The modelled fecundity *ρ* includes all contributions to juvenile survival that occur before the juveniles are independent. Therefore, nestling mortality and early juvenile mortality are included in *ρ*. Fecundity was further assumed to be density-dependent, decreasing exponentially with the ratio *n*_*i*_*b*_*i*_ of local population density *n*_*i*_ and local resource availability 1/*b*_*i*_ [47]. Resource availability was expressed as the overall strength of demographic density-dependence 1/*b*, modulated by the local suitability *h*_*i*_, as $b_{i}=b/h_{i}*100$. This local suitability was determined from habitat preferences provided by Storchová & Horák [48], which were used to determine habitat classes that coarsely delineate the typical habitat of each species. These habitat classes were mapped to CORINE land cover classes (see electronic supplementary material, table A.2), yielding binary habitat maps at a resolution of 100 m. Then, the maps were spatially aggregated to a resolution of 1 km by counting the number of 100 m^2^-habitat cells located within each 1 km^2^-landscape cell *i*. The resulting index ranges from 0 to 100 and was used as suitability index *h*_*i*_. A 10 km buffer around the Swiss border was retained to reduce boundary effects. To summarize, climatic variables determined all three demographic rates while habitat suitability only influenced the density-dependence effect on fecundity.

The process of natal dispersal was modelled in three explicit stages: emigration, transfer and settlement. The emigration probability *p*_*e*_ described the probability with which a juvenile embarks on a dispersal event. To identify the destination cell after transfer, an exponential dispersal kernel with mean dispersal distance $\bar{d}$ and uniformly distributed direction was evaluated individually. If the destination was a suitable cell, the juvenile settled in it. Else, if one of the eight directly neighbouring cells was suitable, the individual settled there and otherwise died. Hence, juveniles suffered a dispersal mortality that was additive to the annual mortality *s*_*j*_. The order of processes in each simulation year was first reproduction, then dispersal, and lastly survival. We used stochastic initial conditions for each model run by drawing from an auto-regressive distribution model [49] that was fitted to the Atlas data of the period 1993–1996.

We estimated the parameters of each DCR simultaneously with all other model parameters (1/*b*, *p*_*e*_, $\bar{d}$) inversely from MHB data using Bayesian inference [50]. As priors for the climate-independent parameters and the intercepts of the DCRs we assumed Gaussian distributions with means and standard deviations derived from the demographic traits provided by Storchová & Horák [48] and the dispersal traits provided by Fandos *et al*. [51] (electronic supplementary material, table A.1). For the DCR coefficients, we used mildly regularizing truncated normal priors with *β*_*m*,*n*_ = 0 ± 1 and truncations at [− 5, 5]. To link model predictions to observations, we compared the simulated abundance of adult breeding females with the observed breeding pair counts. For this, we assumed a negative-binomial likelihood with a truncated Gaussian prior of *σ* = 50 ± 50, bounded between 0 and 500, on the dispersion parameter. To reduce the stochasticity in the likelihood stemming from the non-deterministic nature of the IBM, all counts were spatially aggregated to larger cells of (25 km)^2^ and 20 IBM replicate runs were averaged for each sample. Posterior distributions were estimated using Markov chain Monte Carlo (MCMC) sampling with a differential evolution sampler (DEzs; [52]) implemented by the R package BayesianTools [53]. We ran three independent DEzs chains per species-specific calibration. Each chain had a length of 180 000 iterations, of which the first half was discarded as burn-in period. The MCMCs converged for 8 out of the 9 selected species. Based on this, the goldcrest was excluded from further analysis.

### (c) Evaluation and analyses

To validate and evaluate model predictions, we examined both the full IBM simulations and the extracted DCRs. All evaluations were based on a sample of 400 draws taken from the joint posterior of each species. The full simulations provided spatio-temporal projections of adult abundance, which were used to validate the model fit to MHB counts and to the Swiss breeding bird index [54].

**Figure 2.**
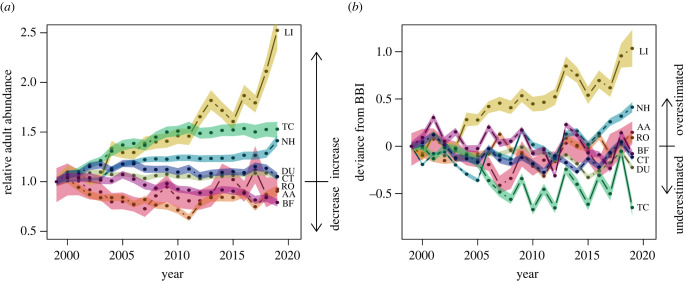
Time series of simulated relative adult abundance (*a*) and their deviation from the Swiss breeding bird index (BBI, *b*), with 1999 (the first year of MHB data) as reference year. Shown are the median and the 80% credible interval. BF, bullfinch (pink); CT, crested tit (dark blue); TC, Eurasian treecreeper (dark green); NH, Eurasian nuthatch (light blue); DU, dunnock (light green); LI, common linnet (yellow); RO, ring ouzel (orange); AA, alpine accentor (red). Electronic supplementary material, figure A.1 shows the 95% credible intervals.

The goodness-of-fit of spatio-temporal projections was evaluated with RMSE (root mean squared error; electronic supplementary material, table A.3) and Harrell’s c-index ([55]; table 2). The c-index is a rank correlation index that generalizes the AUC index to non-binary response variables and we used its implementation in the Hmisc R package [56]. It quantifies the probability that the ranking of a pair of model-projected abundances matches the respective ranking of MHB counts in a given site and year. We henceforth refer to this measure as spatio-temporal c-index. For further validation, we generated model projections of total adult abundance time series relative to the year 1999 (figure 2; electronic supplementary material, figure A.1, left panel). They were compared to the Swiss breeding bird index, which directly estimates the same quantity from MHB data and was thus considered as reference (figure 2; electronic supplementary material, A.1, right panel). Additionally, we quantified spatial and temporal prediction accuracy separately with a spatial AUC and a temporal c-index. These followed the procedures presented in Briscoe *et al.* [35], who compared correlative species distribution models (SDMs) and dynamic occupancy models (DOMs) for 69 Swiss breeding bird species. Specifically, we compressed the abundance predictions to presence–absence data and averaged these across replicate runs to obtain per-cell occupancy probabilities. We then computed the spatial AUC achieved for each year and averaged these across all years to obtain a mean yearly spatial AUC. The temporal c-index (called temporal AUC in [35]) was calculated by comparing the model projections of abundance time series relative to the year 1999 with the Swiss breeding bird index [35]. We used both spatial AUC and temporal c-index for a direct comparison with the performances of the SDMs and DOMs presented in Briscoe *et al*. [35].

**Table 2.** Model evaluation results for each species (column 1). The spatio-temporal c-index (a rank correlation index, column 2) measures prediction accuracy in terms of discriminatory power. Column 3 states the low-density growth rate at median predictor values and columns 4 to 8 give its partial response to the observed trend in each climate variable. Details are given in the methods section. Colours code for strength of response, where grey denotes little to no effect (less than 5% change), light red/blue denote small decrease/increase (more than 5% change), and dark red/blue denote strong decrease/increase (more than 10% change) in growth rate. The last column gives the ratio of simulated adult abundance under scenarios of observed and missing climate change. Given are the median and 80% credible interval.

			*T*_*br*_	*P*_*br*_	*T*_*at*_	*T*_*wn*_	*P*_*wn*_	abund. change
absolute change	c-index	r_med_	+1.0 K	−11.6 mm	+1.1 K	+1.5 K	+29 mm	
bullfinch	0.80	1.20	0.93	1.00	0.96	1.02	0.89	0.85 (0.83–0.90)
crested tit	0.75	1.05	0.94	0.97	0.97	1.01	0.98	0.80 (0.79–0.80)
E. treecreeper	0.77	0.84	1.02	1.00	0.97	1.01	0.98	0.94 (0.90–0.99)
E. nuthatch	0.74	0.56	1.07	1.04	1.00	1.03	1.10	1.07 (1.07–1.08)
dunnock	0.73	1.32	0.97	0.90	0.96	1.00	1.02	0.90 (0.89–0.91)
common linnet	0.72	1.43	1.03	1.05	0.97	1.01	1.10	1.24 (1.24–1.25)
ring ouzel	0.82	1.05	0.95	1.00	1.07	1.02	1.21	1.13 (1.10–1.20)
alpine accentor	0.88	1.05	0.95	0.97	1.00	0.99	1.04	1.10 (1.05–1.13)

**Figure 3.**
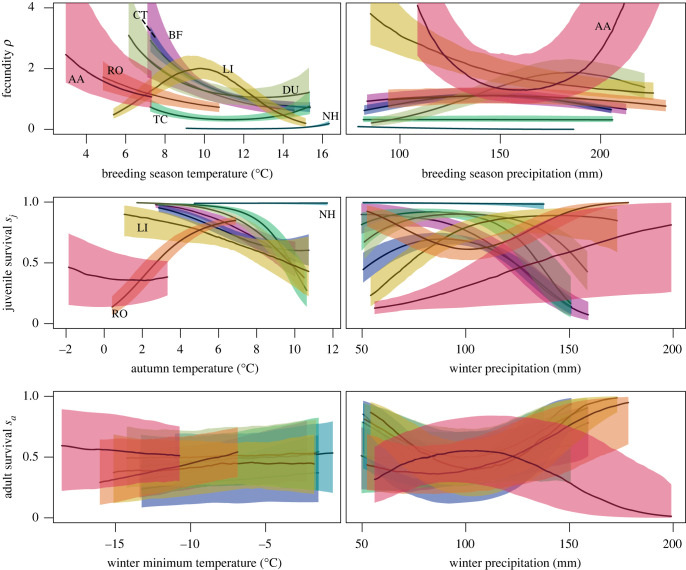
Demography–climate relationships (DCRs) for all species, evaluated over their respective typical climatic ranges. Shown are the median and 80% credible interval. BF, bullfinch (pink); CT, crested tit (dark blue); TC, Eurasian treecreeper (dark green); NH, Eurasian nuthatch (light blue); DU, dunnock (light green); LI, common linnet (yellow); RO, ring ouzel (orange); AA, alpine accentor (red). Electronic supplementary material, figure A.2 shows the single DCRs with both 80% and 95% credible intervals.

As a second step, we visually inspected the conditional response curves of all six DCRs within the range of observed climatic predictors for each species. This range spanned the 10th and 90th percentile of climate values occupied by a species, while the respective second predictor is held constant at its median. We then extracted the median and credible intervals for each demographic rate, as shown in figure 3 and electronic supplementary material, figure A.2. The relationship for fecundity included density-dependence and thus had an additional parameter (1/*b*), describing the strength of density-dependence, and two additional predictors (local habitat suitability *h*_*i*_ and density *n*_*i*_). For deriving the response curves, *h*_*i*_ was held constant at its species-specific median and *n*_*i*_ was set to one breeding pair per 1 km^2^ cell, yielding the fecundity that is realized at low densities.

Lastly, we quantified the effects on predicted population performance that could be attributed to climatic trends over the observed time period. From the three demographic rates, we derived a local, low-density growth rate *r* as an overall measure of population performance. It was given by the leading eigenvalue of the transition matrix (as the population-based equivalent to our IBM), $r=(s_{a}/2)+{\left((s_{a}^{2}/4)+s_{j}\;\rho \right)}^{-(1/2)}$. As a measure of vulnerability to climate change, we assessed the partial response of *r* to the observed changes in each climate variable individually (table 2). For this, we compared a base growth rate, given as the value predicted at the median value of all predictors, with those for which a single predictor was changed. The amount of considered climatic change was determined from the linear trend in each predictor, accumulated over the 20 years of survey data (1999–2019). Thus, the vulnerability indicator combines the effects from both the sensitivity of the growth rate to climate change and the amount of exposure. However, it takes into account only the isolated effect from one predictor, while there are likely interactions between the impacts of different aspects of climate change. As an overall indicator of climate change impact, that simultaneously considers all predictors as well as their interactions, we used the full IBM to generate abundance projections under two scenarios: the factual, observed climate scenario and a counter-factual no-climate-change scenario, which consisted of trend-corrected predictors to simulate a stationary climate. The impact measure was then given by the ratio of projected mean adult abundance over the years 2017–2019 under the scenarios of actual versus stationary climate.

## 3. Results

The goodness-of-fit and discriminatory power of the posterior predictions was assessed using RMSE and the spatio-temporal c-index for all species. The RMSE ranged from 1.3 to 5.7 (electronic supplementary material, table A.3). The spatio-temporal c-index ranged from 72% to 88% (table 2), indicating moderate to excellent model fit depending on the species. The two measures ranked models in a different order but the model for alpine accentor scored best in both. Separately considering the spatial and temporal predictive accuracy (electronic supplementary material, table A.3) showed that all models had good to excellent accuracy in the spatial predictions, as the spatial AUC ranged from 74% to 89%, but less and more variable temporal predictive accuracy, with the temporal c-index ranging from 42% to 80% (electronic supplementary material, table A.3). The IBM projections of total adult abundance are shown in figure 2*a*, relative to the initial year when the MHB was launched (1999). The models for linnet, treecreeper and nuthatch predicted increasing trends, while stable or slightly declining trends were predicted for the remaining species. Those three models also showed the largest deviations from the Swiss breeding bird index, which indicated under-predicted abundance for the treecreeper and over-predicted abundances for linnet and nuthatch (figure 2*b*). Interestingly, the temporal c-index, which considers the local abundance time series at all sites instead of the total Swiss abundance time series, is highest for these three models. Comparing to the SDMs and DOMs presented in Briscoe *et al.* [35], our IBMs showed lower spatial predictive accuracy in terms of spatial AUC (electronic supplementary material, table A.3). By contrast, the IBMs exhibited consistently higher temporal predictive accuracy than SDMs in terms of temporal c-index. Considering DOMs, our IBMs improved the temporal c-index by more than 20% for nuthatch and linnet, and achieved similar or slightly lower temporal predictive accuracy for the other species (electronic supplementary material, table A.3).

The conditional response plots of all six DCRs are shown in figure 3, with the three response rates in rows and their respective temperature and precipitation predictors in columns (separate DCRs per species are shown in electronic supplementary material, figure A.2). For each species, the DCRs were evaluated over their core occupied climatic range (given as the central 80% quantiles), while the respective second predictor is held constant at its median. The fecundity–temperature DCR indicated a trend of lower fecundity with increasing breeding season temperatures. Most species showed a monotonically decreasing relationship, though for the treecreeper and nuthatch, it was almost constant, and only the linnet exhibited a clear unimodal response with an optimum at around 10°C. The fecundity–precipitation DCR showed weak unimodal responses for bullfinch, crested tit and ring ouzel with optima around 150 mm, and a pronounced unimodal response for the dunnock with an optimum around 190 mm. Surprisingly, for the alpine accentor this relationship was bimodal (two optima at the boundaries of a steep inverted parabola), which is physiologically implausible. The DCRs for juvenile survival exhibited high values around 6°C mean autumn temperature for all species apart from the alpine accentor and around 100 mm total winter precipitation for all species apart from the alpine accentor and linnet. We found a monotonically decreasing response of juvenile survival to mean autumn temperature for most species except the nuthatch and ring ouzel. Its response to winter precipitation was strong and monotonically decreasing for the bullfinch, unimodal for the crested tit, treecreeper and dunnock, and monotonically increasing for the linnet and alpine accentor. The ring ouzel exhibited a bimodal response. The DCRs for adult survival were overall weaker and more uncertain than those for fecundity and juvenile survival. The relationship between adult survival and minimum winter temperature was fitted with only a first-order polynomial (table 1) and results indicated a weak but consistent positive trend for most species. Only the alpine accentor, a typical mountain bird, exhibited a constant or slightly decreasing relationship with temperature, which may be attributed to its adaptation to low temperatures. The DCR of adult survival with winter precipitation showed slight bimodal responses for the crested tit, nuthatch, dunnock and linnet that could be deemed physiologically implausible. The alpine accentor showed a unimodal response with an optimum at around 100 mm, and bullfinch, treecreeper and ring ouzel showed slightly increasing adult survival with winter precipitation. We discuss possible explanations for these physiologically implausible DCRs and give remarks on their interpretation in the discussion.

**Figure 4.**
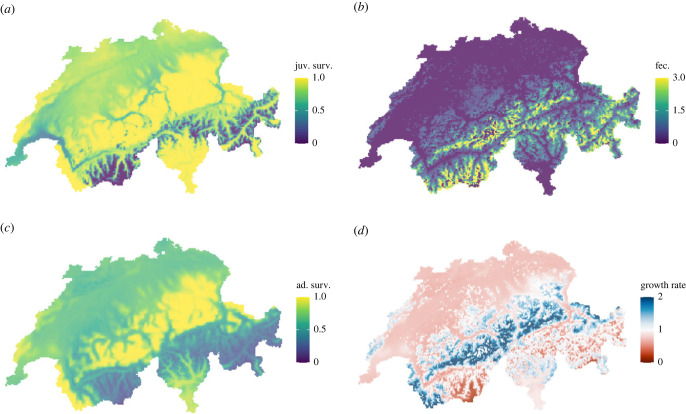
The three demographic rates, juvenile survival (*a*), fecundity (*b*), adult survival (*c*), and the resulting growth rate at low densities (*d*) for the ring ouzel in year 2018. In (*d*), red/blue shades indicate growth rates below/above unity (which is the value associated with a stable population).

To better understand the interplay of the different demographic processes, we summarized the three demographic rates obtained from the DCRs to a density-dependent growth rate *r*. A species can persist only in regions where the climatic conditions allow for sufficient growth. Figure 4*a*–*c* shows illustrative maps of each demographic rate and figure 4*d* presents the resulting growth rate at low density (i.e. at one breeding pair per 1 km^2^) for the ring ouzel in 2018. The map of growth rates highlights areas of negative population growth (*r* < 1) and those of positive growth (*r* > 1), thus providing an assessment of demographically suitable areas. For example, in the northern Alps all three demographic rates largely coincided in exhibiting high values, which resulted in positive growth of ring ouzel. By contrast, some areas in the southern Alps showed high fecundity but low survival probabilities, which resulted in a negative population growth.

To assess the impact of the observed climate change on the study populations, we examined two measures of population response (table 2). First, we calculated the change in low-density growth rate caused by the change of individual climate predictors that was observed over the survey period (second row in table 2). According to this analysis, the most influential climate predictors were the mean temperature during breeding season, *T*_*br*_, which tended to decrease population growth, and precipitation during winter, *P*_*wn*_, which tended to increase population growth. The bullfinch and crested tit were consistently disadvantaged by the change in climate predictors, while the nuthatch benefitted. Other species experienced mixed effects from the changed climate predictors on their growth rates, such as alpine accentor and ring ouzel which were adversely affected by the observed trends in *T*_*br*_, but gained from increases in *P*_*wn*_. Secondly, we compared abundance predictions for the years 2017–2019 under the scenarios of observed climate versus no-climate change (last column in table 2). According to this analysis, some species showed lower current abundances than would have been expected under a no-climate change scenario (bullfinch, crested tit, treecreeper, dunnock). The remaining species exhibited higher predicted current abundances (nuthatch, linnet, ring ouzel, alpine accentor) indicating that these species profited from climate change. Presumably, this benefit was primarily driven by increased winter precipitation. However, a predicted positive effect of climate change on abundance did not necessarily result in increasing populations. For example, the ring ouzel and alpine accentor showed slight negative trends even under the more favourable scenario of observed climate change (figure 2).

## 4. Discussion

Spatio-temporal changes in species abundance result from a complex interplay of multiple ecological processes and environmental drivers. In this study, we analysed the range and population dynamics of Swiss breeding birds using spatially explicit IBMs that included demographic and dispersal processes. We used a generic modelling framework and Bayesian calibration to jointly infer these processes and their DCRs from survey data. This procedure allowed us to disentangle the effects of recent climate change on observed range and population dynamics and to determine if population trends were positively or negatively affected. Yet, results also indicated that care must be taken when interpreting the fitted DCRs as they need to be investigated closely for physiological plausibility. Implausible response shapes can indicate missing or misrepresented ecological processes, missing environmental predictors, or a lack of data to confidently separate the processes. Overall, our framework shows great promise for fitting process-based models to survey data and for attributing observed population trends to ecological and environmental processes [57]. It thus helps improving our mechanistic understanding of range dynamics under climate change and pinpointing missing knowledge.

The increasing availability of long-term monitoring data allows fitting and validating complex process-based population models [35,58]. In this study, we inversely parameterized IBMs based on long-term, nationwide survey data in Switzerland. Importantly, our framework extends previous approaches [29] by allowing higher flexibility in the representation of demographic processes. Our models explicitly included crucial factors such as density-dependence and climatic impacts on demographic rates [11]. At the same time, simplifying assumptions were made to obtain parsimonious models that were able to broadly capture the observed abundance patterns. This included describing the transfer phase with a dispersal kernel and employing a relatively simple stage-structured, female-only population model [59]. Modelling only the female part of the population was justified by assuming that reproduction is not limited by adult male abundance, considering that the modelled species breed in pairs and birds show a balanced or even male-skewed adult sex ratio [60]. Apart from this, a female-only model does not explicitly consider Allee effects and can be thus expected to perform better for populations with high rather than low densities. The presented framework, however, can accommodate other modelling assumptions than those exemplified here, since it is generic and flexible. It uses openly available tools and can thus be readily applied to other populations.

When validating our spatio-temporal predictions against observations, our models achieved moderate to excellent predictive accuracy, with the highest performance for the alpine accentor. We also evaluated the ability of our models to predict the adult abundance time-series against the independently derived Swiss breeding bird index and found adequate fit of the temporal dynamics for most species. We compared our results to the findings of a recent study by Briscoe *et al.* [35], that evaluated the performance of correlative SDMs and DOMs for predicting range dynamics of Swiss breeding birds. DOMs represent local occurrence as a result from colonization and extinction dynamics, but do not explicitly consider dispersal. Briscoe *et al.* [35] found that SDMs made more reliable spatial predictions, but DOMs provided superior temporal predictions of occupancy dynamics. Our results corroborate and generalize these findings, showing that dynamic population models which explicitly consider demography and dispersal can provide more accurate predictions of range and population dynamics than other currently used approaches. Still, SDMs provided better spatial predictions of occupancy and our IBMs improved the temporal predictions over DOMs only for a few but not all species. In part, this can be explained by the fact that our IBMs used fewer predictors and instead imposed structure through specific assumptions on the causal demographic–ecological processes. Its predictions are therefore expected to fit less closely to training data and in turn perform better on extrapolation. Further, the underlying heuristic habitat maps were based only on published trait data of main habitat preferences and may be overestimating the amount of suitable habitat. The preferences were given in terms of coarse habitat classes, such as deciduous or coniferous forest, while the true habitat preferences may be much more specific and met in only a smaller subset of the identified habitat areas. Lower predictive accuracy compared to DOMs could also be related to structural uncertainty in our models. For example, the fecundity DCRs of nuthatch and treecreeper were mostly constant in both predictors such that all spatial variation in fecundity originated from the habitat maps, possibly indicating missing environmental predictors. Overall, our results demonstrate that dynamic models that are sufficiently flexible to reflect main abundance patterns can improve predictions of range dynamics.

The calibrated DCRs allow investigation into how demographic processes vary with climate. When interpreting the individual DCRs, it is important to consider the specific role of the respective demographic rate within the model. In our IBMs, juvenile and adult survival rates represent primarily winter and autumn mortality but do not account for dispersal mortality. The latter is instead represented by failed dispersal events and was modelled as climate-independent. Thus, effective annual survival is lower than suggested by the DCRs and resulting local population dynamics alone. Further, fecundity described the number of independent juveniles produced per breeding pair and year. It therefore subsumes factors such as the number of broods per year and early juvenile mortality. Most fitted DCRs showed monotonous or unimodal relationships. For example, fecundity decreased with above-median temperatures during breeding season for all species. Maximum juvenile survival was surprisingly high, approaching 100% for some species. This seems unrealistic even under favourable climatic conditions as juveniles of most species typically experience higher mortality in their first winter compared to adults. In the context of our model structure, however, high apparent juvenile survival rates could be compensating for high simulated dispersal mortality. Thus, in order to compare survival rates of our models against empirical estimates, the number of juveniles lost during dispersal needs to be discounted. With respect to parameter uncertainty, DCRs of fecundity and juvenile survival were more certain and had smaller credible intervals than DCRs for adult survival. This suggests that fecundity and juvenile survival indeed depended strongly on climate, while adult survival may be mediated by additional processes.

Explicit consideration of demographic responses to environmental factors can improve our mechanistic understanding of species niche and range dynamics [22,29]. Despite the advantages of mechanistic models, however, they are often difficult to parameterize. Fortunately, inverse parametrization can make use of commonly available data such as abundance and occurrence data. Here, all DCRs were calibrated to observed abundance data, and population dynamics emerged from the combined effect of the three demographic processes together with dispersal. Their interactions can entail limited identifiability of parameters from the available data, if the same abundance patterns arise from different combinations of survival and fecundity values. This increases the uncertainty of parameter estimates [61], leading to uncertain DCRs, and additional data may be needed for successful calibration. Further, structural model error can be present where the modelled processes do not correspond to the true ecological processes. In such cases, the calibration may compensate for flawed modelling assumptions and lead to unexpected DCR shapes [61]. The inversely fitted DCRs are therefore still phenomenological and their transferability should be interpreted carefully. For instance, some fitted DCRs were bimodal with two optima at the extremes of their climatic range. This was found for the fecundity–precipitation DCR of the alpine accentor and the juvenile survival–precipitation DCR of the ring ouzel. Such a relationship is physiologically implausible as demographic rates typically peak within certain climatic limits. A possible explanation for the alpine accentor is that the assumed fecundity–habitat relationship may not hold and the DCR attempts to compensate for low realized fecundities at the range margins, where low habitat suitability is projected. With respect to the ring ouzel, the local winter conditions may be poor predictors of survival since large parts of their Swiss population migrates during winter. Also, for most species, the relationships of adult survival with winter precipitation was slightly bimodal. This can be a result from interactions among adult survival and juvenile survival, which shared minimum winter temperature as a common predictor. These two DCRs tended to be negatively correlated for most species, apart from ring ouzel and alpine accentor, which supports this hypothesis. Such a negative correlation was also found by Dybala *et al*. [62], who modelled the impact of climate change on the demography of song sparrows in California. Tavecchia *et al.* [63] showed that climate-driven vital rates do not necessarily imply climate-driven population dynamics, especially in highly mobile species, as trade-offs can mask the changes in underlying processes. Further, an example for a potential missing mechanism are negative species interactions [64], which can confound the value of a DCR, especially towards the range margins. Therefore, we advise that extrapolation to non-analogue climatic conditions outside the sampled range of training data should only be attempted if the fitted DCRs appear plausible. Importantly, despite limitations in interpretability and transferability of individual DCRs, their combined effects within the full model simulations were still able to reproduce the observed abundances and yield moderate to excellent model predictions. Thereby, DCRs facilitate an improved mechanistic understanding of the underlying processes and potentially missing information, which offers substantial advantages over simple hybrid models [29,30].

Our presented model framework allows complex assessments of the factors and mechanisms that underlie model predictions. By combining the demographic rates to an overall growth rate, we can better understand the causes for demographic suitability of different geographical regions and compare interpretations across different model frameworks. For example, we found that the ring ouzel was predicted to generally persist in high altitudes where fecundity is high enough, but that some of these high-altitude areas are climatically unsuitable due to high winter mortality. According to Barras *et al.* [44], future losses in abundance are expected especially in the northern Alps, where we predicted highest current growth rates, and gains in abundance are expected in the central Alps, where we predicted lowest current growth rates. To assess the impacts of observed climatic changes on the demographic performance of the studied species, we considered two measures of climate vulnerability. First, evaluating the conditional impact of each climatic predictor identified breeding-season temperature and winter precipitation as the most influential variables. This impact is a combined effect of the magnitude of already observed climate change and the sensitivity of the growth rate to them. However, more targeted investigations are needed to understand the biological pathways by which a given abiotic factor controls an ecological process. For example, Barras *et al.* [65] explained for ring ouzel that elevated temperatures during breeding negatively impacted the feeding of chicks by parents. Indeed, our model confirms a negative effect of breeding-season temperature on growth rate for this species. Also, our predicted current growth rate (1.05) coincides with the one measured by Barras *et al.* [66] (1.04). Second, comparing the simulated abundances under observed climate with a no-climate change scenario, we were able to estimate the degree to which predicted abundance trends could be attributed to recent climate change. Such analyses are useful to understand the overall direction of the combined effects of climate change on populations. For example, ring ouzel and alpine accentor show slightly declining population trends, both in the breeding bird index and in our projections. However, our models indicated that predicted abundance was still higher then in the no-climate change scenario, implying that both species would experience even stronger population declines without recent climate change. Overall, our models suggested that typical mountain species tend to benefit from recent climate change (without necessarily amounting to positive population trends), while lowland species are already negatively affected by climate change. We do not expect the positive effects for mountain species to last far into the future, as climatic changes become more pronounced. In fact, while we assessed the effect of contemporary climate change only, the vulnerability of Swiss breeding birds to future climate change was investigated previously by comparing current climatic conditions with those in 2100 [67]. According to these predictions, alpine accentor and ring ouzel will suffer strong range contractions until the end of this century. Here, correctly identified DCRs can help to identify promising avenues for effective management. By explicitly investigating how individual demographic rates are affected by climate change, targeted measures can be designed to support these vulnerable processes and mitigate climate change effects.

Given the broad impact of climate change on ecological processes, we urgently need better predictions. Our study demonstrates that calibrating detailed, process-based models from survey data is feasible and can improve predictions of spatio-temporal range and population dynamics over other modelling approaches. Further, explicitly modelling the responses of separate demographic rates to climate allows the development of a mechanistic understanding of the differential effects of contemporary climate change. Yet, it is important to acknowledge that our model framework relies on confining modelling assumptions and inverse parameterization. It is, therefore, still phenomenological to a certain extent and the resulting DCRs need to be interpreted cautiously. Specifically, they should be inspected for plausibility before making predictions into the future or drawing conclusions for targeted conservation management. For future research, we advocate a stronger integration of experimentally or empirically measured DCRs, for example by adding such information as strong informative prior in the Bayesian calibration. In order to determine appropriate model structures for modelling climate change responses, more abiotic and biotic mechanisms should be tested, for example by considering additional environmental predictors and alternative model structures. Ultimately, this will increase our confidence for drawing conclusions on the mechanisms underlying complex range and population dynamics and making predictions into the future.

## Data Availability

The study uses data from the Swiss breeding bird survey and the Swiss breeding bird index provided by the Swiss ornithological institute, Sempach. All scripts and data required to run the presented analyses can be accessed from the public GitHub repository https://github.com/UP-macroecology/Malchow_DemogEnv_2022 or from the Zenodo archive with doi:10.5281/zenodo.7830083 [68]. The used R packages are open-source software. RangeShiftR is available from GitHub. For this study, a tagged development version was used that is available at: https://github.com/RangeShifter/RangeShiftR-package/releases/tag/v.1.1-beta.0. BayesianTools is available directly from CRAN or from GitHub under: https://github.com/florianhartig/BayesianTools. The appendix is provided in electronic supplementary material [69].

## References

[1] Parmesan C, Yohe G. 2003 A globally coherent fingerprint of climate change impacts across natural systems. Nature **421**, 37-42. (10.1038/nature01286) 12511946

[2] Hooper DU et al. 2012 A global synthesis reveals biodiversity loss as a major driver of ecosystem change. Nature **486**, 105-108. (10.1038/nature11118) 22678289

[3] Rosenzweig C, Neofotis P. 2013 Detection and attribution of anthropogenic climate change impacts. WIREs Clim. Change **4**, 121-150. (10.1002/wcc.209)

[4] Chen IC, Hill JK, Ohlemüller R, Roy DB, Thomas CD. 2011 Rapid range shifts of species associated with high levels of climate warming. Science **333**, 1024-1026. (10.1126/science.1206432) 21852500

[5] Thompson PL et al. 2022 Groundfish biodiversity change in northeastern Pacific waters under projected warming and deoxygenation. Phil. Trans. R. Soc. B **378**, 20220191. (10.1098/rstb.2022.0191) PMC10225861 37246387

[6] Martay B, Brewer MJ, Elston DA, Bell JR, Harrington R, Brereton TM, Barlow KE, Botham MS, Pearce-Higgins JW. 2017 Impacts of climate change on national biodiversity population trends. Ecography **40**, 1139-1151. (10.1111/ecog.02411)

[7] Gregory RD et al. 2022 Drivers of the changing abundance of European birds at two spatial scales. Phil. Trans. R. Soc. B **378**, 20220198. (10.1098/rstb.2022.0198) PMC10225857 37246375

[8] Urban MC et al. 2016 Improving the forecast for biodiversity under climate change. Science **353**, aad8466. (10.1126/science.aad8466) 27609898

[9] Fei S, Desprez JM, Potter KM, Jo I, Knott JA, Oswalt CM. 2017 Divergence of species responses to climate change. Sci. Adv. **3**, e1603055. (10.1126/sciadv.1603055) 28560343 PMC5435420

[10] Urban MC et al. 2022 Coding for life: designing a platform for projecting and protecting global biodiversity. BioScience **72**, 91-104. (10.1093/biosci/biab099)

[11] Ehrlén J, Morris WF. 2015 Predicting changes in the distribution and abundance of species under environmental change. Ecol. Lett. **18**, 303-314. (10.1111/ele.12410) 25611188 PMC4674973

[12] Zurell D, König C, Malchow AK, Kapitza S, Bocedi G, Travis J, Fandos G. 2022 Spatially explicit models for decision-making in animal conservation and restoration. Ecography **2022**, 05787. (10.1111/ecog.05787)

[13] Guisan A, Thuiller W. 2005 Predicting species distribution: offering more than simple habitat models. Ecol. Lett. **8**, 993-1009. (10.1111/j.1461-0248.2005.00792.x) 34517687

[14] Cuddington K, Fortin MJ, Gerber LR, Hastings A, Liebhold A, O’Connor M, Ray C. 2013 Process-based models are required to manage ecological systems in a changing world. Ecosphere **4**, art20. (10.1890/ES12-00178.1)

[15] Connolly SR, Keith SA, Colwell RK, Rahbek C. 2017 Process, mechanism, and modeling in macroecology. Trends Ecol. Evol. **32**, 835-844. (10.1016/j.tree.2017.08.011) 28919203

[16] Briscoe NJ et al. 2019 Forecasting species range dynamics with process-explicit models: matching methods to applications. Ecol. Lett. **22**, 1940-1956. (10.1111/ele.13348) 31359571

[17] Evans TG, Diamond SE, Kelly MW. 2015 Mechanistic species distribution modelling as a link between physiology and conservation. Conserv. Physiol. **3**, cov056. (10.1093/conphys/cov056) 27293739 PMC4778482

[18] Franklin J. 2010 Moving beyond static species distribution models in support of conservation biogeography. Divers. Distribut. **16**, 321-330. (10.1111/j.1472-4642.2010.00641.x)

[19] Singer A, Schweiger O, Kühn I, Johst K. 2018 Constructing a hybrid species distribution model from standard large-scale distribution data. Ecol. Modell. **373**, 39-52. (10.1016/j.ecolmodel.2018.02.002)

[20] Weber MM, Stevens RD, Diniz-Filho JAF, Grelle CEV. 2017 Is there a correlation between abundance and environmental suitability derived from ecological niche modelling? A meta-analysis. Ecography **40**, 817-828. (10.1111/ecog.02125)

[21] Thuiller W et al. 2014 Does probability of occurrence relate to population dynamics? Ecography **37**, 1155-1166. (10.1111/ecog.00836) 25722536 PMC4338510

[22] Schurr FM et al. 2012 How to understand species’ niches and range dynamics: a demographic research agenda for biogeography. J. Biogeogr. **39**, 2146-2162. (10.1111/j.1365-2699.2012.02737.x)

[23] Paniw M et al. 2021 The myriad of complex demographic responses of terrestrial mammals to climate change and gaps of knowledge: a global analysis. J. Anim. Ecol. **90**, 1398-1407. (10.1111/1365-2656.13467) 33825186

[24] Housset JM, Carcaillet C, Girardin MP, Xu H, Tremblay F, Bergeron Y. 2016 *In situ* comparison of tree-ring responses to climate and population genetics: the need to control for local climate and site variables. Front. Ecol. Evol. **4**, 00123. (10.3389/fevo.2016.00123)

[25] Treurnicht M, Pagel J, Esler KJ, Schutte-Vlok A, Nottebrock H, Kraaij T, Rebelo AG, Schurr FM. 2016 Environmental drivers of demographic variation across the global geographical range of 26 plant species. J. Ecol. **104**, 331-342. (10.1111/1365-2745.12508)

[26] Cianfrani C, Satizábal HF, Randin C. 2015 A spatial modelling framework for assessing climate change impacts on freshwater ecosystems: response of brown trout (*Salmo trutta* L.) biomass to warming water temperature. Ecol. Modell. **313**, 1-12. (10.1016/j.ecolmodel.2015.06.023)

[27] Bonnot TW, Cox WA, Thompson FR, Millspaugh JJ. 2018 Threat of climate change on a songbird population through its impacts on breeding. Nat. Clim. Change **8**, 718-722. (10.1038/s41558-018-0232-8)

[28] Dunham KD, Tucker AM, Koons DN, Abebe A, Dobson FS, Grand JB. 2021 Demographic responses to climate change in a threatened Arctic species. Ecol. Evol. **11**, 10 627-10 643. (10.1002/ece3.7873) PMC8328435 34367602

[29] Pagel J, Schurr FM. 2012 Forecasting species ranges by statistical estimation of ecological niches and spatial population dynamics. Global Ecol. Biogeogr. **21**, 293-304. (10.1111/j.1466-8238.2011.00663.x)

[30] Zurell D et al. 2016 Benchmarking novel approaches for modelling species range dynamics. Glob. Change Biol. **22**, 2651-2664. (10.1111/gcb.13251) PMC4972146 26872305

[31] Railsback SF, Grimm V. 2019 Agent-based and individual-based modeling: a practical introduction, 2nd edn. Princeton, NJ: Princeton University Press.

[32] DeAngelis DL, Mooij WM. 2005 Individual-based modeling of ecological and evolutionary processes. Annu. Rev. Ecol. Evol. Syst. **36**, 147-168. (10.1146/annurev.ecolsys.36.102003.152644)

[33] Bocedi G, Palmer SC, Malchow AK, Zurell D, Watts K, Travis JM. 2021 RangeShifter 2.0: an extended and enhanced platform for modelling spatial eco-evolutionary dynamics and species’ responses to environmental changes. Ecography **44**, 1453-1462. (10.1111/ecog.05687)

[34] Malchow AK, Bocedi G, Palmer SCF, Travis JMJ, Zurell D. 2021 RangeShiftR: an R package for individual-based simulation of spatial eco-evolutionary dynamics and species’ responses to environmental changes. Ecography **44**, 1443-1452. (10.1111/ecog.05689)

[35] Briscoe NJ, Zurell D, Elith J, König C, Fandos G, Malchow AK, Kéry M, Schmid H, Guillera-Arroita G. 2021 Can dynamic occupancy models improve predictions of species’ range dynamics? A test using Swiss birds. Glob. Change Biol. **27**, 4269-4282. (10.1111/gcb.15723) PMC13420806 34037281

[36] Maggini R, Lehmann A, Kéry M, Schmid H, Beniston M, Jenni L, Zbinden N. 2011 Are Swiss birds tracking climate change? Detecting elevational shifts using response curve shapes. Ecol. Modell. **222**, 21-32. (10.1016/j.ecolmodel.2010.09.010)

[37] Gobiet A, Kotlarski S, Beniston M, Heinrich G, Rajczak J, Stoffel M. 2014 21st century climate change in the European Alps—a review. Sci. Total Environ. **493**, 1138-1151. (10.1016/j.scitotenv.2013.07.050) 23953405

[38] Ceppi P, Scherrer SC, Fischer AM, Appenzeller C. 2012 Revisiting Swiss temperature trends 1959–2008. Int. J. Climatol. **32**, 203-213. (10.1002/joc.2260)

[39] Karger DN, Conrad O, Böhner J, Kawohl T, Kreft H, Soria-Auza RW, Zimmermann NE, Linder HP, Kessler M. 2017 Climatologies at high resolution for the Earth’s land surface areas. Sci. Data **4**, 170122. (10.1038/sdata.2017.122) 28872642 PMC5584396

[40] Karger DN, Conrad O, Böhner J, Kawohl T, Kreft H, Soria-Auza RW, Zimmermann NE, Linder HP, Kessler M. 2018 Data from: Climatologies at high resolution for the earth’s land surface areas. *EnviDat*. (10.16904/envidat.228.v2.1) PMC5584396 28872642

[41] European Union. 2022 *Copernicus Land Monitoring Service*. https://land.copernicus.eu/.

[42] Schmid H, Zbinden N, Keller V. 2004 Überwachung der Bestandsentwicklung häufiger Brutvögel in der Schweiz. Sempach, Switzerland: Schweizerische Vogelwarte.

[43] Schmid H, Luder R, Naef-Daenzer B, Graf R, Zbinden N. 1998 Schweizer Brutvogelatlas 1993–1996. Sempach, Switzerland: Schweizerische Vogelwarte.

[44] Barras AG, Braunisch V, Arlettaz R. 2021 Predictive models of distribution and abundance of a threatened mountain species show that impacts of climate change overrule those of land use change. Diver. Distribut. **27**, 989-1004. (10.1111/ddi.13247)

[45] Saether BE, Bakke O. 2000 Avian life history variation and contribution of demographic traits to the population growth rate. Ecology **81**, 642-653. (10.1890/0012-9658(2000)081[0642:ALHVAC]2.0.CO;2)

[46] Cox WA, Thompson III FR, Reidy JL, Faaborg J. 2013 Temperature can interact with landscape factors to affect songbird productivity. Glob. Change Biol. **19**, 1064-1074. (10.1111/gcb.12117) 23504884

[47] Neubert MG, Caswell H. 2000 Density-dependent vital rates and their population dynamic consequences. J. Math. Biol. **41**, 103-121. (10.1007/s002850070001) 11039693

[48] Storchová L, Horák D. 2018 Life-history characteristics of European birds. Glob. Ecol. Biogeogr. **27**, 400-406. (10.1111/geb.12709)

[49] Dormann CF et al. 2007 Methods to account for spatial autocorrelation in the analysis of species distributional data: a review. Ecography **30**, 609-628. (10.1111/j.2007.0906-7590.05171.x)

[50] Hartig F, Calabrese JM, Reineking B, Wiegand T, Huth A. 2011 Statistical inference for stochastic simulation models—theory and application. Ecol. Lett. **14**, 816-827. (10.1111/j.1461-0248.2011.01640.x) 21679289

[51] Fandos G, Talluto M, Fiedler W, Robinson RA, Thorup K, Zurell D. 2023 Standardised empirical dispersal kernels emphasise the pervasiveness of long-distance dispersal in European birds. J. Anim. Ecol. **92**, 158-170. (10.1111/1365-2656.13838) 36398379

[52] ter Braak CJF, Vrugt JA. 2008 Differential evolution Markov Chain with snooker updater and fewer chains. Stat. Comput. **18**, 435-446. (10.1007/s11222-008-9104-9)

[53] Hartig F, Minunno F, Paul S. 2019 BayesianTools: general-purpose MCMC and SMC samplers and tools for Bayesian statistics. *R package version 0.1.7*.

[54] Knaus P, Strebel N, Sattler T. 2022 The State of Birds in Switzerland 2022. Sempach, Switzerland: Swiss Ornithological Institute.

[55] Newson R. 2006 Confidence intervals for rank statistics: Somers’ d and extensions. Stata J. **6**, 309-334. (10.1177/1536867X0600600302)

[56] Tikhonov G, Opedal ØH, Abrego N, Lehikoinen A, de Jonge MM, Oksanen J, Ovaskainen O. 2020 Joint species distribution modelling with the R-package Hmsc. Methods Ecol. Evol. **11**, 442-447. (10.1111/2041-210X.13345) 32194928 PMC7074067

[57] Gonzalez A, Chase JM, O’Connor MI. 2022 A framework for the detection and attribution of biodiversity change. Phil. Trans. R. Soc. B **378**, 20220182. (10.1098/rstb.2022.0182) PMC10225858 37246383

[58] Fordham DA, Bertelsmeier C, Brook BW, Early R, Neto D, Brown SC, Ollier S, Araújo MB. 2018 How complex should models be? Comparing correlative and mechanistic range dynamics models. Glob. Change Biol. **24**, 1357-1370. (10.1111/gcb.13935) 29152817

[59] Caswell H. 2000 Matrix population models, vol. 1. Sunderland, MA: Sinauer.

[60] Donald PF. 2007 Adult sex ratios in wild bird populations. Ibis **149**, 671-692. (10.1111/j.1474-919X.2007.00724.x)

[61] Oberpriller J, Cameron DR, Dietze MC, Hartig F. 2021 Towards robust statistical inference for complex computer models. Ecol. Lett. **24**, 1251-1261. (10.1111/ele.13728) 33783944

[62] Dybala KE, Eadie JM, Gardali T, Seavy NE, Herzog MP. 2013 Projecting demographic responses to climate change: adult and juvenile survival respond differently to direct and indirect effects of weather in a passerine population. Glob. Change Biol. **19**, 2688-2697. (10.1111/gcb.12228) 23606580

[63] Tavecchia G, Tenan S, Pradel R, Igual JM, Genovart M, Oro D. 2016 Climate-driven vital rates do not always mean climate-driven population. Glob. Change Biol. **22**, 3960-3966. (10.1111/gcb.13330) 27279167

[64] Schultz EL et al. 2022 Climate-driven, but dynamic and complex? A reconciliation of competing hypotheses for species’ distributions. Ecol. Lett. **25**, 38-51. (10.1111/ele.13902) 34708503

[65] Barras AG, Niffenegger CA, Candolfi I, Hunziker YA, Arlettaz R. 2021 Nestling diet and parental food provisioning in a declining mountain passerine reveal high sensitivity to climate change. J. Avian Biol. **52**, e02649. (10.1111/jav.02649)

[66] Barras AG, Blache S, Schaub M, Arlettaz R. 2021 Variation in demography and life-history strategies across the range of a declining mountain bird species. Front. Ecol. Evol. **9**, 780706. (10.3389/fevo.2021.780706)

[67] Maggini R et al. 2014 Assessing species vulnerability to climate and land use change: the case of the Swiss breeding birds. Diver. Distribut. **20**, 708-719. (10.1111/ddi.12207)

[68] Malchow A-K, Hartig F, Reeg J, Kéry M, Zurell D. 2023 Demography–environment relationships improve mechanistic understanding of range dynamics under climate change (1.0.0). *Zenodo*. (10.5281/zenodo.7830083) PMC10225853 37246385

[69] Malchow A-K, Hartig F, Reeg J, Kéry M, Zurell D. 2023 Demography–environment relationships improve mechanistic understanding of range dynamics under climate change. Figshare. (10.6084/m9.figshare.c.6620248) PMC10225853 37246385

